# Association between obesity and clinical prognosis in patients infected with SARS-CoV-2

**DOI:** 10.1186/s40249-020-00703-5

**Published:** 2020-06-29

**Authors:** Shao-Hang Cai, Wei Liao, Shu-Wei Chen, Li-Li Liu, Si-Yao Liu, Zhi-Dan Zheng

**Affiliations:** 1 Department of Infectious Diseases and Hepatology Unit, Nanfang Hospital, Southern Medical University, Guangzhou, Guangdong Province China; 2 Intensive Care Unit, Sun Yat-sen University Cancer Center, Guangzhou, Guangdong Province China; 3 Collaborative Innovation Center of Cancer Medicine, Guangzhou, Guangdong Province China; 4 Department of Head and Neck Surgery, Sun Yat-sen University Cancer Center, Guangzhou, Guangdong Province China; 5 Department of Pathology, Sun Yat-sen University Cancer Center, Guangzhou, Guangdong Province China; 6 Emergency Department, First Affiliated Hospital of Xiamen University, Xiamen, Fujian province China; 7 Department of Infectious Diseases, Dongguan people’s Hospital, Southern Medical University, Dongguan, Guangdong Province China

**Keywords:** SARS-CoV-2, Obesity, Body mass index, Prognosis, Pneumonia, Acute respiratory distress syndrome

## Abstract

**Background:**

It is well established that obesity is a disease of sustained low-grade inflammation. However, it is currently unknown if obesity plays a role in the clinical manifestations and prognosis of severe acute respiratory syndrome coronavirus 2 (SARS-CoV-2) infected patients. In this study, we aimed to investigate whether obesity played a role in clinical manifestations and prognosis in patients infected with SARS-CoV-2.

**Methods:**

This is a retrospective multicenter clinical study. A total of 96 patients hospitalized with SARS-CoV-2 infection were enrolled from Dongguan People’s Hospital, Nanfang hospital and the First Affiliated Hospital of Xiamen University between 23 January and 14 February 2020. Demographic and clinical data were extracted from medical records. Acute respiratory distress syndrome (ARDS) was defined as oxygenation index (PaO_2_/FiO_2_) ≤ 300 mmHg. We grouped patients through the body mass index (BMI). Associations were examined using the *t* test, *χ*^2^ test and multivariate logistic forward regression test.

**Results:**

Patients with BMI <  24 were significantly younger (*P* = 0.025) with lower creatine kinase (*P* = 0.013), lower diastolic pressure blood (*P* = 0.035), lower serum creatinine (*P* = 0.012), lower lactate dehydrogenase (*P* = 0.001) and higher platelet count (*P* = 0.002). The BMI level was 20.78 ± 3.15 in patients without pneumonia compared with the patients with pneumonia (23.81 ± 3.49, *P* = 0.001). For patients without ARDS, an average BMI level of 22.65 ± 3.53 was observed, significantly lower than patients with ARDS (24.57 ± 3.59, *P* = 0.022). The mean BMI was 22.35 ± 3.56 in patients experienced with relieving the clinical symptoms or stable condition by radiographic tests, lower than patients with disease exacerbation with 24.89 ± 3.17 (*P* = 0.001). In addition, lymphocyte count (r = − 0.23, *P* = 0.027) and platelet count (r = − 0.44, *P* < 0.001) were negatively correlated with BMI. While hemoglobin (r = 0.267, *P* = 0.008), creatine kinase (r = 0.331, *P* = 0.001), serum creatinine (r = 0.424, *P* < 0.001) and lactate dehydrogenase (r = 0.343, *P* = 0.001) were significantly positive correlated with BMI. Multivariate analysis showed that older age (*OR* = 1.046, *P* = 0.009) and BMI ≥ 24 (*OR* = 1.258, *P* = 0.005) were independent risk factors associated ICU admission while BMI ≥ 24 (*OR* = 4.219, *P* = 0.007) was independent risk factor associated with radiographic disease exacerbation.

**Conclusions:**

Our study found BMI was significantly associated with clinical manifestations and prognosis of patients with SARS-CoV-2 infection. For patients with increased risk, clinicians should intervene promptly to avoid disease progression.

## Background

A series of unexplained viral pneumonia cases occurred worldwide recently [1]. Subsequent studies have shown that this series of pneumonia was associated with a new coronavirus infection (SARS-CoV-2) [1, 2]. This virus epidemic is posing a huge threat to global public health [3–5].

This sudden infectious disease is mainly manifested as fever, fatigue, and cough [6, 7]. Upper respiratory symptoms are relatively rare, which may be due to the fact that the virus infects cells through angiotensin converting enzyme 2, which is mainly expressed in cells of the lower respiratory tract [8, 9]. About one-half of the patients developed dyspnoea after 1 week [6]. In severe and critical cases, it progressed rapidly (average 9 days) to acute respiratory distress syndrome (ARDS) with only mild symptoms in early stage [6, 10]. This brings difficulties in managing the infectious diseases. In order to control the epidemic better and reduce the spread of the disease, early detection, quarantine and timely treatment are the keys to management. However, it is still no very clear which cohort of the population is at high risk.

Obesity is now a global health issue [11, 12]. It is well established that obesity is a disease of sustained low-grade inflammation [13, 14]. Such inflammation has been suggested to be associated with obesity related disease. A series of inflammatory markers have been proved related with both obesity and obesity associated disease. Previous research has confirmed a positive association between obesity and C-reactive protein level [15]. Similar associations have also been reported for erythrocyte sedimentation rate [16] and some other inflammatory cytokines [17, 18]. Those findings further support the potential association between obesity and inflammation. The interactions between obesity and infectious diseases have recently received increasing recognition. Previously published data have indicated an association between obesity and poor outcome in pandemic H1N1 influenza infection [19]. Obesity is an established risk factor for surgical-site infections, nosocomial infections, periodontitis and skin infections [20]. However, it is currently unknown if obesity plays a role in the clinical manifestations and prognosis of SARS-CoV-2 infected patients.

In this study, the clinical manifestations and clinical outcomes of SARS-CoV-2 infected patients were evaluated. The purpose of this study was to determine the role of obesity in the prognoses of patients infected with SARS-CoV-2.

## Subjects and methods

### Subjects

This is a retrospective multicentre clinical study. This study was approved by the institutional ethics board of Nanfang Hospital, Southern Medical University. All consecutive patients with confirmed SARS-CoV-2 infection in Dongguan People’s Hospital, Nanfang hospital affiliated Southern Medical University and the First Affiliated Hospital of Xiamen University between 23 January and 14 February 2020 were enrolled. Oral consent was obtained from patients. All patients were diagnosed with SARS-CoV-2 by pharyngeal swab samples. The SARS-CoV-2 infection diagnostic standard is detection of two target genes of SARS-CoV-2 in pharyngeal swab samples using polymerase chain reaction (PCR) [10].

### Data collection

We collected patient medical records and recorded patient demographic and clinical data. All data were reviewed by a team of experienced physicians. ARDS was defined as acute onset, oxygenation index (PaO_2_/FiO_2_) ≤ 300 mmHg, and a chest radiograph that showed patchy shadows [21]. We categorized patients based on body mass index (BMI, kg/m^2^). All BMIs were calculated based on the height and weight measured on admission.

### Statistical analysis

Continuous variables were expressed as average values and compared with student *t*-test. The categorical variables were expressed as a number (percentage) and compared by chi-square test. A univariate and multivariate regression analysis was used, with the results presented as an odds ratio (*OR*) with a 95% confidence interval (*CI*). All analyses were performed using SPSS software package (version 13.0, SPSS Inc. Chicago, USA), alpha level was 0.05.

## Results

### Characteristics of patients infected SARS-CoV-2 grouped by BMI

A total of 96 patients infected with SARS-CoV-2 were enrolled. Among these, 59 had a BMI <  24 and 37 of them had a BMI ≥ 24. Demographic and clinical characteristics were compared and shown in Table 1. Patients with BMI < 24 were significantly younger than others (*P* = 0.025), while the creatine kinase (CK) (*P* = 0.013), diastolic pressure blood (DBP), serum creatinine (*P* = 0.012) and lactate dehydrogenase (LDH) (*P* = 0.001) level were significantly lower. Platelet counts (*P* = 0.002) were significantly higher in patients with BMI < 24.

**Table 1:** The demographics and clinical characteristics between patients with COVID-19

Characteristic	Group		***P*** value
	BMI < 24	BMI ≥ 24	
Sample size, *n*	59	37	–
Sex (male), *n* (%)	31 (52.5)	23 (62.2)	0.355
Age (years)	35.41 ± 18.45	44.00 ± 17.23	0.025
Systolic blood pressure	124.84 ± 16.11	126.29 ± 15.51	0.666
Diastolic blood pressure	81.13 ± 9.61	85.57 ± 10.08	0.035
Creatine kinase	84.04 ± 112.12	169.37 ± 212.25	0.013
Serum lactic acid	1.64 ± 0.70	1.39 ± 0.65	0.123
Neutrophil count	3.33 ± 1.50	3.31 ± 1.41	0.927
Lymphocyte count	1.46 ± 1.04	1.19 ± 0.61	0.169
Hemoglobin	139.22 ± 15.07	142.51 ± 19.95	0.361
Platelet count	222.23 ± 66.42	180.89 ± 51.92	0.002
Serum creatinine	60.58 ± 19.09	70.19 ± 15.18	0.012
ALT	20.38 ± 16.65	26.22 ± 18.51	0.118
AST	21.70 ± 8.59	26.05 ± 13.88	0.065
Lactate dehydrogenase	177.82 ± 54.39	221.94 ± 73.21	0.001
Smoking tobacco, *n* (%)	5 (8.5)	3 (8.1)	0.950

*ALT* Alanine aminotransferase; *AST* Aspartate aminotransferase

We also analyzed the difference of clinical characteristics between patients with age < 18 and ≥ 18 years old. We found that patients younger than 18 years (*n* = 15) had lower systolic blood pressure (SBP) and DBP than adults (*n* = 81) (SBP: 114.67 ± 12.98 vs 127.01 ± 15.62 mmHg, *P* = 0.011; DBP: 75.25 ± 8.52 vs 84.02 ± 9.73 mmHg, *P* = 0.004). The average level of lymphocyte count in patients younger than 18 years was 2.29 ± 1.63, which was significantly higher than that of ≥ 18-year age group (1.18 ± 0.56, *P* < 0.001). However, no significantly different with CK level and lactate level was found between the two groups.

### Association between BMI and clinical outcomes of SARS-CoV-2 infected patients

To further evaluate the association between BMI and the clinical outcomes of SARS-CoV-2 infected patients, we measured the proportion of patients with different outcomes (see Table 2). Among 96 patients infected with SARS-CoV-2, 21 of them were without pneumonia based on the finding of computerized tomography (CT) scan, while 75 patients were diagnosed with pneumonia. The proportions of patients with negative CT results were 85.7, 14.3 and 0% for patients with BMI <  24, 24–27.9 and ≥ 28, respectively; however, in pneumonia group, the proportions were 54.7, 32.0 and 13.3% in respective BMI groups (*P* = 0.027). Mean BMI value was 20.78 ± 3.15 in patients without pneumonia, compared with 23.81 ± 3.49 with pneumonia (*P* = 0.001).

**Table 2:** Proportion of viral pneumonia by groups

Variable	Patients with COVID-19		***P*** value
	Without pneumonia *n* = 21, *n* (%)	With pneumonia *n* = 75, *n* (%)	
BMI stage			0.027
< 24	18 (85.7)	41 (54.7)	
24–27.9	3 (14.3)	24 (32.0)	
≥ 28	0 (0)	10 (13.3)	
BMI level	20.78 ± 3.15	23.81 ± 3.49	0.001

Among the whole cohort, 25 patients were diagnosed with ARDS. The proportions of patients with ARDS with a BMI < 24, 24–27.9, and ≥ 28 were 52.0, 24.0, and 24.0%, respectively, which were significantly different from the proportions of 64.8, 29.6 and 5.6% in patients without ARDS (*P* = 0.035). Average BMI was 22.65 ± 3.53 in patients without ARDS, which was significantly lower than that of patients with ARDS (24.57 ± 3.59, *P* = 0.022, Table 3).

**Table 3:** Proportion of ARDS by groups

Variable	Patients with COVID-19		***P*** value
	Without ARDS *n* = 71, *n* (%)	With ARDS *n* = 25, *n* (%)	
BMI stage			0.035
< 24	46 (64.8)	13 (52.0)	
24–27.9	21 (29.6)	6 (24.0)	
≥ 28	4 (5.6)	6 (24.0)	
BMI level	22.65 ± 3.53	24.57 ± 3.59	0.022

Patients enrolled were received at least one repeat CT scan within 1 month. After treatment, 66 patients showed improved or stable disease, while 30 showed exacerbated disease. The mean BMI was 22.35 ± 3.56 in patients with disease stable or relief, which was lower than the mean BMI of 24.89 ± 3.17, present in patients with disease exacerbation (*P* = 0.001), as shown in Table 4.

**Table 4:** Proportion of clinical outcome by groups

Variables	COVID-19 patients with disease		***P*** value
	Stable-relieve *n* = 66, *n* (%)	Exacerbation *n* = 30, *n* (%)	
BMI stage			0.001
< 24	49 (74.2)	10 (33.3)	
24–27.9	13 (19.7)	14 (46.7)	
≥ 28	4 (6.1)	6 (20.0)	
BMI level	22.35 ± 3.56	24.89 ± 3.17	0.001

### Correlation between BMI and clinical variables

**Fig. 1:**
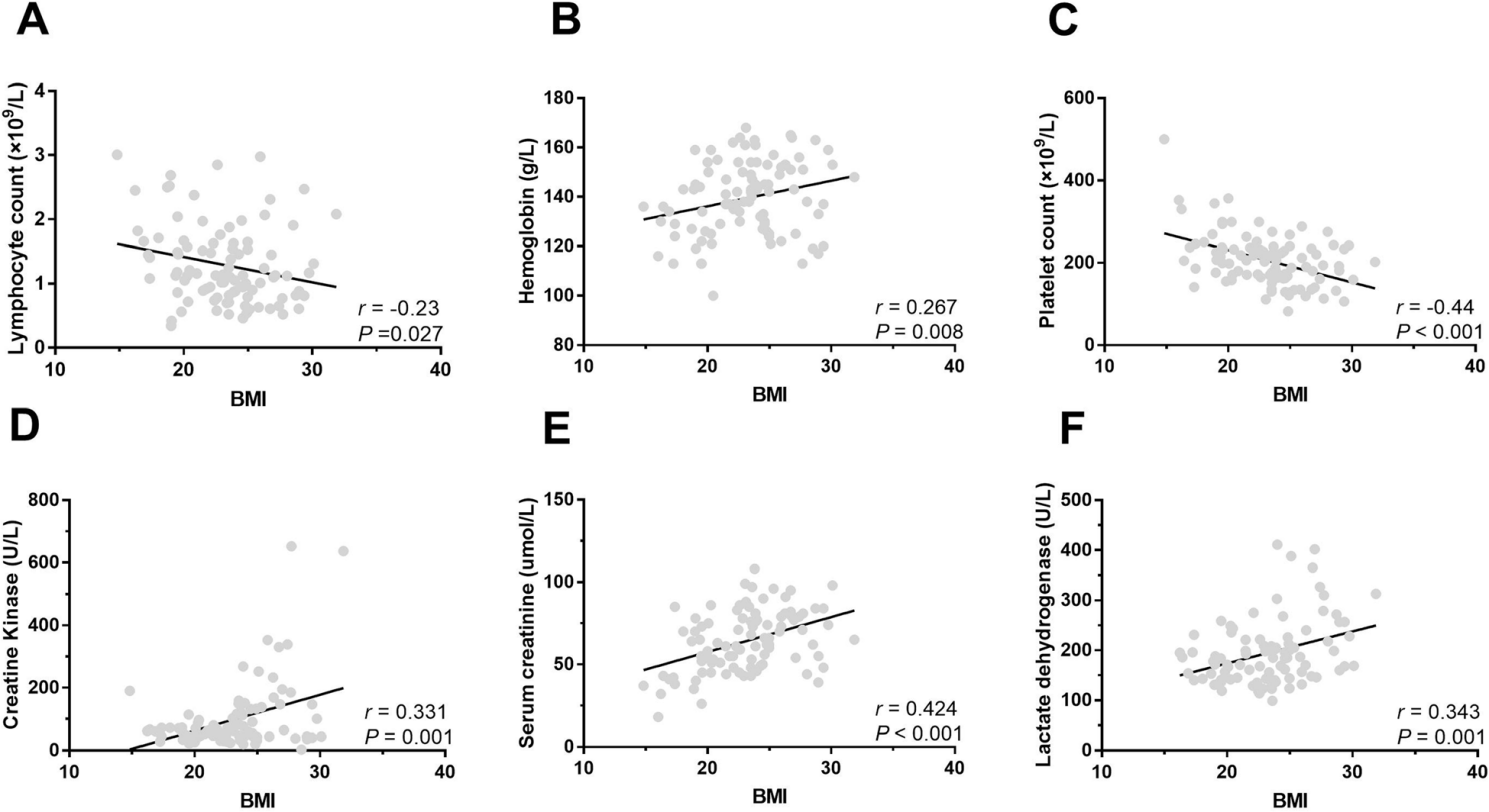
Correlation between BMI and clinical variables in SARS-CoV-2 infected patients. **a** Correlation between lymphocyte and BMI (r = − 0.23, *P* = 0.027). **b** Correlation between haemoglobin and BMI (r = 0.267, *P* = 0.008). **c** Correlation between platelet count and BMI (r = − 0.44, *P* < 0.001). **d** Correlation between CK level and BMI (r = 0.331, *P* = 0.001). **e** Correlation between serum creatinine and BMI (r = 0.424, *P* < 0.001). **f** Correlation between LDH and BMI (r = 0.343, *P* = 0.001). BMI, Body mass index. CK, creatine kinase. LDH, lactate dehydrogenase

Interestingly, we found several unexpected clinical variables that were significantly correlated with BMI in all enrolled SARS-CoV-2 infected patients (Fig. 1). Lymphocyte count (r = − 0.23, *P* = 0.027) and platelet count (r = − 0.44, *P* < 0.001) were negatively correlated with BMI, while haemoglobin (r = 0.267, *P* = 0.008), CK (r = 0.331, *P* = 0.001), serum creatinine (r = 0.424, *P* < 0.001), and LDH (r = 0.343, *P* = 0.001) were significantly positive correlated with BMI.

### Univariate and multivariate analysis of factors associated with ICU admission

Logistic regression was performed to identify factors that were significantly associated with ICU admission in SARS-CoV-2-infected patients. The results of this multivariate analysis indicated that older age (*OR* = 1.046, *P* = 0.009) and BMI ≥ 24 (*OR* = 1.258, *P* = 0.005) were independent risk factors associated with ICU admission among patients with SARS-CoV-2 infection (Table 5).

**Table 5:** Factors associated with ICU admission

Variables	Univariate analysis			Multivariate analysis		
	***OR***	95% ***CI***	***P*** value	***OR***	95% ***CI***	***P*** value
Sex (male vs female)	1.361	0.595–3.112	0.465			
Age	1.043	1.017–1.070	0.001	1.046	1.011–1.081	0.009
Systolic blood pressure	1.026	0.998–1.055	0.065			
Diastolic blood pressure	1.023	0.980–1.068	0.295			
Creatine kinase	1.002	0.999–1.004	0.256			
Serum lactic acid	0.956	0.496–1.843	0.894			
Neutrophil count	0.919	0.689–1.227	0.569			
Lymphocyte count	0.532	0.274–1.032	0.062			
Hemoglobin	1.016	0.992–1.040	0.198			
Platelet count	0.992	0.985–1.000	0.041			
Serum creatinine	1.021	0.998–1.045	0.073			
ALT	1.019	0.995–1.044	0.117			
AST	1.042	1.002–1.084	0.042			
Lactate dehydrogenase	1.009	1.002–1.016	0.016			
Smoking tobacco (yes vs no)	1.041	0.234–4.630	0.958			
BMI level (< 24 vs ≥ 24)	5.190	2.110–12.763	< 0.001	1.258	1.071–1.478	0.005

*OR* Odds ratio; *CI* Confidence interval; *ALT* Alanine aminotransferase; *AST* Aspartate aminotransferase

### Univariate and multivariate analysis of factors associated with radiographic disease exacerbation

We also conducted logistic regression to identify factors that were associated with radiographic disease exacerbation in SARS-CoV-2 infected patients. Multivariate analyses showed that BMI ≥ 24 (*OR* = 4.219, *P* = 0.007) was independent risk factors associated with SARS-CoV-2 infected patients who experienced radiographic disease exacerbation (Table 6).

**Table 6:** Factors associated with radiographic exacerbation

Variables	Univariate analysis			Multivariate analysis		
	***OR***	95% ***CI***	***P*** value	***OR***	95% ***CI***	***P*** value
Sex (male vs female)	1.025	0.429–2.448	0.956			
Age	1.023	0.998–1.048	0.069			
Systolic blood pressure	1.009	0.982–1.038	0.513			
Diastolic blood pressure	1.001	0.958–1.045	0.978			
Creatine kinase	1.002	1.000–1.005	0.097			
Serum lactic acid	1.041	0.523–2.074	0.909			
Neutrophil count	0.979	0.725–1.322	0.889			
Lymphocyte count	0.684	0.350–1.338	0.267			
Hemoglobin	1.006	0.981–1.032	0.630			
Platelet count	0.997	0.990–1.004	0.398			
Serum creatinine	1.008	0.984–1.033	0.503			
ALT	1.017	0.992–1.042	0.190			
AST	1.025	0.986–1.065	0.208			
Lactate dehydrogenase	1.001	0.994–1.008	0.756			
Smoking tobacco (yes vs no)	1.356	0.302–6.083	0.691			
BMI level (< 24 vs ≥ 24)	5.765	2.255–14.734	< 0.001	4.219	1.490–11.944	0.007

*OR* Odds ratio; *CI* Confidence interval; *ALT* Alanine aminotransferase; *AST* Aspartate aminotransferase

## Discussion

In this study, we found that obesity played an important role in development of COVID-19. SARS-CoV-2 infected patients with a higher BMI were more likely to develop ARDS and to experience exacerbated disease. Moreover, we found that patients with higher BMIs had lower lymphocyte counts, lower platelet counts, and higher levels of haemoglobin, CK, creatinine, and LDH. Our results may help to stratify patients with SARS-CoV-2 infection: patients with high BMIs should receive prompt intervention to avoid disease progression. Most patients with SARS-CoV-2 infection will develop pneumonia [22]. However, a small number of patients have negative imaging findings for unclear reasons. As we know, the largest study to-date indicated that 3498 of the 3665 (95.5%) confirmed cases were diagnosed with pneumonia [23]. According to the 2019 New Coronavirus Pneumonia Diagnosis and Treatment Plan recommended by the National Health Committee of China, these patients without pneumonia showed only low fever and mild fatigue, and usually recovered after 1 week. Our research confirmed that, although SARS-CoV-2 infection was confirmed, some patients had negative CT results. In addition, our study found that this situation was more likely to occur in young patients with lower BMIs. Although the prognosis of these patients seems to be better than older patients with high BMIs, repeated CT examinations are still needed, particularly if the clinical symptoms worsen.

Obesity is a common worldwide epidemic. Obesity is usually accompanied by a low-grade chronic inflammatory state, which is characterized by an increase in systemic inflammation markers [13, 16]. This mild chronic inflammation and non-specific activation of the immune system are thought to induce obesity-related diseases [13]. The reasons why obesity causes inflammation have not yet been well described, but studies have revealed that adipocytes secrete a variety of cytokines that help initiate an inflammatory response [24, 25]. In long-term chronic inflammation, a series of changes occur in the body, including in blood glucose, lipid, and hormone levels [24]. These changes are accompanied by insulin and catecholamine resistance, abnormal tissue remodelling and fibrosis [24]. Some patients with SARS-CoV-2 infections rapidly developed into critically ill patients, in which case the disease usually manifests as ARDS [6, 26]. Current research showed that the mortality rate of SARS-CoV-2 infected patients was 4–15% [6, 26]. It is therefore important to identify this population early. However, the subpopulations who became severely ill may have had moderate to low fever during the early course of the disease, and these patients are still difficult to screen. Whether obesity plays a role here is unknown, and further researches are still needed.

Our results suggest high BMI is closely related to patient disease progression. At the same time, our study showed that BMI was negatively correlated with lymphocytes and platelets, but positively correlated with CK, LDH, and creatinine. Patients with SARS-CoV-2-infections often experience lymphocytopenia [4, 7]. It is unknown whether SARS-CoV-2 infection will induce immune deficiency, and whether obesity and adipocytes play a role here. However, according to the results of our study, patients who with a high BMI, even if their symptoms are not obvious, should be given sufficient attention to avoid rapid deterioration.

The current study has several limitations. Firstly, this was a retrospective survey, with a relatively limited sample size. In addition, the role of obesity in the pathophysiology of SARS-CoV-2 infection requires further research to confirm. It is also an important topic to identify patients with SARS-CoV-2-associated pneumonia developing ARDS during the course of disease. This issue merits additional research.

## Conclusions

Our study found that BMI was significantly related with clinical manifestations and clinical outcomes of patients with SARS-CoV-2 infections. Patients with higher BMIs were more likely to develop ARDS and to experience disease progression. Older age and high BMI are independent risk factors associated with ICU admission in SARS-CoV-2 infected patients, while patients with higher BMIa are more likely to experience disease exacerbation. For patients with risk factors, clinicians should intervene promptly to avoid disease progression.

## Data Availability

Authors can confirm all relevant data are included in the article and materials are available on request from the authors. The original data of this study has been uploaded to the RDD platform (RDDA2020001502).

## Abbreviations

ARDS: Acute respiratory distress syndrome
BMI: Body mass index
*OR*: Odds ratio
*CI*: Confidence interval
CK: Creatine kinase
DBP: Diastolic pressure blood
SBP: Systolic blood pressure
CT: Computerized tomography
COVID-19: Corona Virus Induced Disease 2019
